# Global Climate Anomalies and Potential Infectious Disease Risks: 2014-2015

**DOI:** 10.1371/currents.outbreaks.95fbc4a8fb4695e049baabfc2fc8289f

**Published:** 2015-01-26

**Authors:** Jean-Paul Chretien, Assaf Anyamba, Jennifer Small, Seth Britch, Jose L. Sanchez, Alaina C. Halbach, Compton Tucker, Kenneth J. Linthicum

**Affiliations:** Division of Integrated Biosurveillance, Armed Forces Health Surveillance Center, Silver Spring, Maryland, USA; Biospheric Sciences Laboratory, NASA Goddard Space Flight Center, Greenbelt, Maryland, USA; Biospheric Sciences Laboratory, NASA Goddard Space Flight Center, Greenbelt, Maryland, USA; Center for Medical, Agricultural, and Veterinary Entomology, USDA Agricultural Research Service, Gainesville, Florida, USA; Division of Global Emerging Infections Surveillance and Response System (GEIS), Armed Forces Health Surveillance Center (AFHSC), Silver Spring, Maryland, USA; Division of Global Emerging Infections Surveillance and Response System (GEIS), Armed Forces Health Surveillance Center (AFHSC), Silver Spring, Maryland, USA; Earth Sciences Division, NASA/Goddard Space Flight Center, Greenbelt, Maryland, USA; Center for Medical, Agricultural, and Veterinary Entomology, USDA Agricultural Research Service, Gainesville, Florida, USA

## Abstract

Background: The El Niño/Southern Oscillation (ENSO) is a global climate phenomenon that impacts human infectious disease risk worldwide through droughts, floods, and other climate extremes. Throughout summer and fall 2014 and winter 2015, El Niño Watch, issued by the US National Oceanic and Atmospheric Administration, assessed likely El Niño development during the Northern Hemisphere fall and winter, persisting into spring 2015. Methods: We identified geographic regions where environmental conditions may increase infectious disease transmission if the predicted El Niño occurs using El Niño indicators (Sea Surface Temperature [SST], Outgoing Longwave Radiation [OLR], and rainfall anomalies) and literature review of El Niño-infectious disease associations. Results: SSTs in the equatorial Pacific and western Indian Oceans were anomalously elevated during August-October 2014, consistent with a developing weak El Niño event. Teleconnections with local climate is evident in global precipitation patterns, with positive OLR anomalies (drier than average conditions) across Indonesia and coastal southeast Asia, and negative anomalies across northern China, the western Indian Ocean, central Asia, north-central and northeast Africa, Mexico/Central America, the southwestern United States, and the northeastern and southwestern tropical Pacific. Persistence of these conditions could produce environmental settings conducive to increased transmission of cholera, dengue, malaria, Rift Valley fever, and other infectious diseases in regional hotspots as during previous El Niño events. Discussion and Conclusions: The current development of weak El Niño conditions may have significant potential implications for global public health in winter 2014-spring 2015. Enhanced surveillance and other preparedness measures in predicted infectious disease hotspots could mitigate health impacts.

## Background

The El Niño/Southern Oscillation (ENSO) is a large-scale ocean-atmosphere phenomenon that involves a warming or cooling of sea surface temperature (SST) across the central and east-central equatorial Pacific Ocean. El Niño and La Niña, the warm and cold phases, respectively, of the ENSO cycle, impact human health across much of the world through increased risk of natural disasters, such as droughts, floods, and tropical cyclones. These in turn affect agricultural yields, cause air pollution due to landscape fires, and enable transmission of various infectious diseases^1^^,^ 2 ^,^ 3.

During the summer and fall of 2014 and winter of 2015, the US National Oceanic and Atmospheric Administration (NOAA) Climate Prediction Center (CPC) issued an El Niño Watch. The most recent estimate of January 8, 2015 assessed an approximately 50-60% chance of El Niño developing during the next 2 months (Northern Hemisphere winter) and persisting into spring 4. There are uncertainties about its evolution, but consensus favors a weak event.

Following a strong El Niño in 1997-1998, US Government organizations and international partners collaborated to initiate a climatological monitoring program as part of a global surveillance network for emerging infectious diseases^5^^,^ 6. This effort provided early warning of public health impacts associated with the 2006-2007 El Niño^7^^,^ 8 ^,^ 9, and issues monthly^10^ and *ad hoc* updates of ENSO assessments and possible ENSO-associated infectious disease risks. Here we draw on recent climatological data, and historical El Niño-health associations, to assess the likelihood of El Niño in coming months. We identify geographic regions where infectious disease transmission may increase if the predicted El Niño occurs.

## Methods

Detailed descriptions of the El Niño indicators and other climatological data used in our program are available elsewhere^7^. We typically express measurements as anomalies, or deviations from the long-term, month-specific or seasonal (3-month) mean. Briefly, the indicators include:

- SST in the NINO3.4 region (eastern equatorial Pacific Ocean; 5°N-5°S, 120°W-170°W), often used as an indicator of the phase and amplitude of ENSO events (El Niño is characterized by five consecutive 3-month running means of SST anomalies in the NINO3.4 region above +0.5°C).
- SST anomalies in the Western Indian Ocean (WIO); warmer NINO3.4 and WIO SSTs indicate a higher likelihood of El Niño.
- Global SSTs (1981-present).
- Outgoing Longwave Radiation (OLR; 1979-present), an indicator of cloudiness (Positive anomalies indicate warm, dry conditions; negative anomalies indicate cool, cloudy conditions and rainfall).
- Precipitation (Global Precipitation Climatology Project; 1979-present).

To identify regions at risk for El Niño-associated infectious disease activity, we used the literature review of Kovats et al.^1 ^and replicated their search strategy in PubMed to identify subsequent publications (May 2002-November 2014). We supplemented these results with subjective assessments of possible El Niño impacts.

## Results

SSTs in the NINO3.4 and WIO regions reached higher-than-normal temperatures in October 2014 (+0.49°C and +0.48°C, respectively, just below the +0.5°C threshold that must persist for an El Niño event to occur) after cooling during the summer.

The tongue-appearing pattern of the SST anomaly off the Peruvian coast, with SST anomalies ≈ +1.5°C along the equator, is typical of a developing El Niño event (Figure 1).

**Sea Surface Temperature (SST) Anomalies, October 2014.**
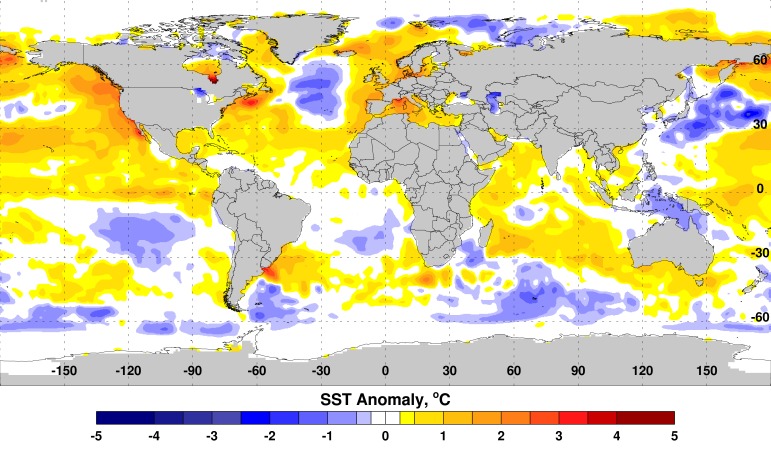
Above-normal SSTs persisted in the equatorial eastern Pacific Ocean along the equator (≈ +1.5°C) and in the equatorial Indian Ocean (≈ +0.5-1.0°C). The tongue-like structure of the SST anomaly off the Peruvian coast is typical of a developing El Nino event.

WIO warming (anomalies ≈ +0.5°C to +1.0°C) off the east African coast also is consistent with previous El Niño events. Anomalous positive SST in the eastern Pacific Ocean drives tropical and extra-tropical convective activity influencing global rainfall and temperature patterns.

The SST anomalies during August-October 2014 are comparable to those preceding the 2006-2007 El Niño, with more extreme elevations in the WIO and less extreme but positive and rising NINO3.4 elevations (Table 1).

**Table 1. Comparison of Western Indian Ocean (WIO) and Eastern Pacific Ocean Region 3.4 (NINO3.4) Sea Surface Temperature Anomalies (°C): 2006-2007 El Nino versus 2014.** 

	2006		2014	
	WIO	NINO3.4	WIO	NINO3.4
August	+0.17	+0.40	-0.00	+0.20
September	+0.09	+0.62	+0.09	+0.45
October	+0.17	+0.78	+0.48	+0.49

Some impacts from the current SST anomaly patterns can be observed in the pattern of global convective activity illustrated by the OLR anomaly patterns. During August through October 2014, large positive departures (>+35 watts per meter squared [W/m^2^]) in OLR across Indonesia and coastal southeast Asia indicate drier than average conditions, while large negative departures (<-40 W/m^2^) across northern China, the western Indian Ocean, central Asia, north-central and northeast Africa, Mexico/Central America, the southwestern United States, and the northeastern and southwestern tropical Pacific suggest wetter than normal conditions (Figure 2).

**Outgoing Longwave Radiation (OLR) Anomalies, August-October 2014.**
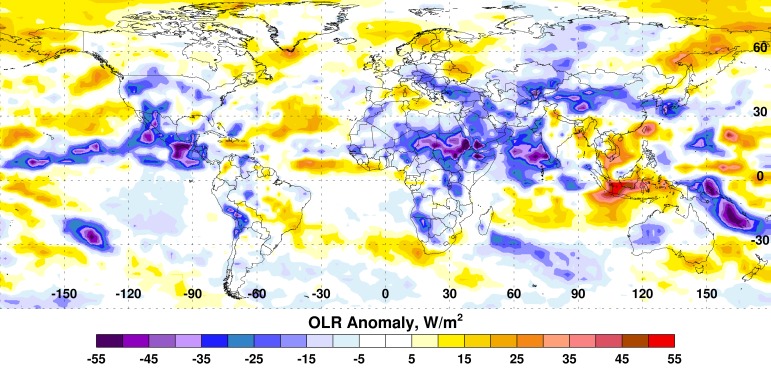
Used to infer tropical precipitation, current OLR anomalies show very dry conditions (brown to red colors) across Indonesia and most of southeast Asia and enhanced precipitation across northern China, the western Indian Ocean, central Asia, north-central and northeast Africa, Mexico/Central America, the southwestern United States, and the northeastern and southwestern tropical Pacific.

Further details of current ENSO conditions are available on the Climate Prediction Center website^4^ and the Rift Valley fever (RVF) Monitor website^10^.

The dramatic shifts in large-scale convective centers around the global tropics during El Niño lead to extreme weather conditions, with above-normal rainfall and flooding in some regions and unusually high temperatures and drought conditions in others (Figure 3).

**Summary Correlation between Monthly NINO3.4 Sea Surface Temperature and Rainfall Anomalies, 1997-2008.**
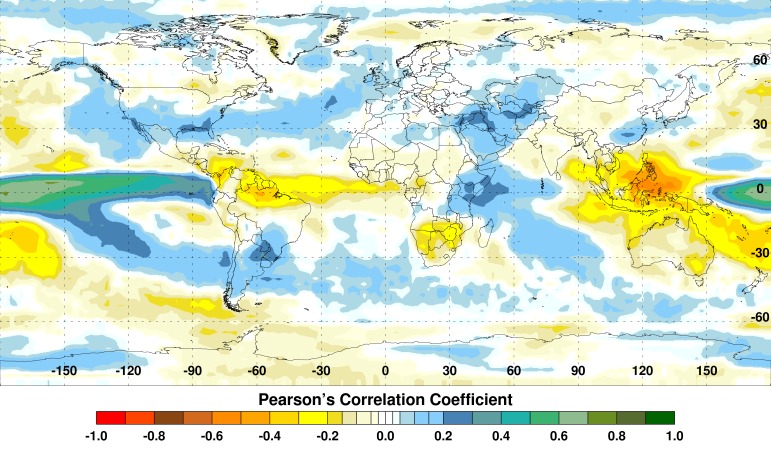
El Niño events are associated with extremes of elevated or depressed rainfall (blue/green or yellow/red, respectively).

Currently, several regions show extreme departures in rainfall, including southeast Asia, northeast Africa, Mexico/Central America, and the southwestern United States (Figure 4).

**Global Seasonal (August-October) Rainfall Anomalies.**
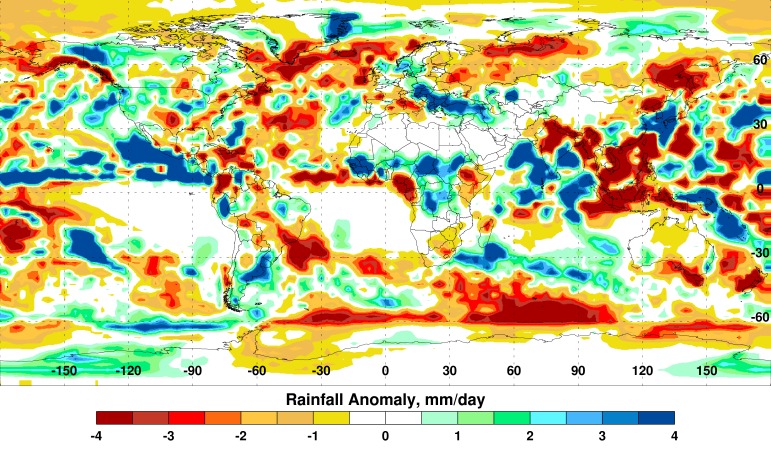
Departures in average rainfall coincide with Outgoing Longwave Radiation anomalies (Fig. 2).

Considering these typical El Niño effects on regional weather and the recent El Niño forecast, there is a high likelihood for drought conditions to prevail in southeast Asia, west-central and southern Africa, northeast Brazil, Ecuador, and Mexico during the next 5 to 8 months; as well as elevated rainfall and flooding in southwest Asia, eastern Africa, coastal Peru and Ecuador, much of the southern portion of South America, and the US southwest, including southern California.

The development of these conditions has important implications for global public health. For each of several diseases, including ones of high global health significance, multiple epidemiological studies have linked El Niño events with increased incidence in human populations (Table 2).

**Table 2. El-Nino-Associated Disease Transmission Enhancement in Human Populations: Examples.** 

Disease	Region	Possible El Nino Effects on Disease Dynamics
Cholera	Africa 11 ^,^ 12 : Great Lakes region; Asia 1 ^,^ 18 ^,^ 19 ^,^ 20 ^,^ 21 ^,^ 22 : Bangladesh, India (coastal), Sri Lanka; South America 45 : Peru	Warmer water temperatures promote bacteria proliferation; flooding causes contamination of water sources, and may increase susceptibility to infection via stress.
Dengue	Asia/Pacific 1 ^,^ 13 ^,^ 14 ^,^ 15 ^,^ 16 ^,^ 17 : Indonesia, Thailand, Pacific Islands, Australia (Queensland); North America 23 ^,^ 24 ^,^ 25 : Mexico, United States (southern); South America 1 ^,^ 26 : Colombia, Ecuador (coastal), French Guiana, Suriname	Dry conditions: Peri-domestic water storage promotes *Aedes aegypti* mosquito vector breeding; elevated temperatures reduce the extrinsic incubation period in *Ae. aegypti* and *Ae. albopictus* vectors; warm, dry conditions may promote vegetation patterns favorable for vector development. Wet conditions: Elevated rainfall promotes *Ae. aegypti* and *Ae. albopictus* breeding.
Hantavirus infection	Asia 27 ^,^ 28 ^,^ 29 ^,^ 30 ^,^ 31 : China (eastern; hemorrhagic fever with renal syndrome); North America 32 : United States (southwestern; hantavirus pulmonary syndrome)	Elevated rainfall increases food availability for rodent reservoirs (vegetation), which expands rodent populations and may promote contact with humans.
Leishmaniasis	Central/South America 1 ^,^ 33 ^,^ 34 ^,^ 35 ^,^ 36 : Brazil (eastern), Costa Rica, Colombia, French Guiana	Warmer temperatures or dry conditions may favor sand fly vectors or contribute to waning human immunity (e.g., via malnutrition or temporarily suppressing disease transmission).
Malaria	Asia 1 ^,^ 37 ^,^ 38 : China (Anhui Province), India/Pakistan (Punjab), Sri Lanka; South America 1 ^,^ 39 ^,^ 40 ^,^ 41 : Colombia, French Guiana, Guyana, Peru (coastal), Venezuela	Elevated rainfall promotes *Anopheles* mosquito vector breeding and survival, and vectorial capacity.
Plague	Africa 43 : Madagascar; North America 44 : United States (western)	Heavy rains increase food availability for populations of susceptible rodents; cooler temperatures may increase infectious flea abundance.
Rift Valley fever	Africa 2 ^,^ 5 : East Africa	Flooding of dry mosquito vector habitats promotes hatching of (transovarially-) infected eggs, and vector breeding and survival.
Respiratory illness	Asia 46 ^,^ 47 : Southeast Asia/Indonesia	Drought may contribute to forest fires, which cause air pollution that may increase risk of respiratory infection.
Ross River virus disease	Asia 1 ^,^ 42 : Australia (Queensland/Murray-Darling River region)	Warm conditions may increase mosquito vector longevity, and thereby vectorial capacity.

Single-study associations have been reported for others, including shigellosis in Bangladesh^18^; hepatitis A in Australia^48^; dysentery in eastern China^49^; and bartonellosis^50^, dermatological infections^51^, and *Vibrio parahaemolyticus* 52 infection in Peru.

El Niño may impact dynamics of other diseases related to ones with demonstrated El Niño associations. For example, chikungunya virus shares vectors with dengue virus (*Aedes aegypti* and *Aedes albopictus* mosquitoes) and often circulates in the same areas. Wet conditions linked to El Niño have been associated with increased dengue incidence on the Mexico-United States (Texas) border^23^ and could enhance chikungunya transmission in the region as well. Both countries have reported imported cases and local transmission since December 2013, when the first, and ongoing, chikungunya epidemic in the Americas began. Florida, the current US focus of local chikungunya and dengue transmission, and other US Gulf Coast states harbor winter populations of chikungunya and dengue virus mosquito vectors. They could experience increased or new transmission with prolonged wet conditions in late 2014 and early 2015. Conversely, warm, dry conditions may increase dengue and chikungunya transmission in other areas where both are endemic (Table 2).

Based on these findings and considerations, we recommend enhanced surveillance and preparedness for specific geographic regions that may be at risk for El Niño-linked infectious disease activity during late 2014 through spring 2015 (Figure 5).

**Potential Infectious Disease Risks Associated with El Nino in 2014-2015.**
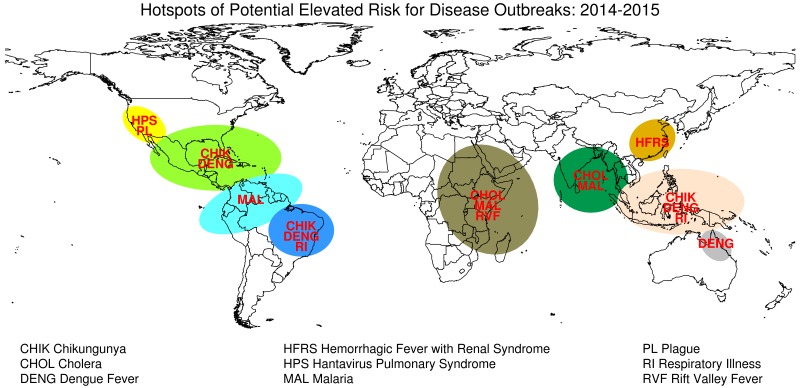

## Discussion and Conclusions

Based on current global climate anomaly conditions and forecasts, El Niño is likely to develop during late 2014 and persist into early 2015. The expected effects on regional weather patterns include persistent high temperatures and drought in some areas, and heavy rainfall and flooding in others. This may enhance populations of particular vectors and the transmission of various infectious diseases in human and animal populations.

Although local weather conditions mediate part of ENSO’s influence on infectious disease transmission (“teleconnections”), incorporating ENSO indicators into disease risk predictions offers advantages. ENSO forecasts typically can anticipate local weather effects several months before they manifest, providing lead-time for public health risk communication, enhancement of disease and vector surveillance programs, provisioning of clinical resources (for example, vaccines and diagnostics), and other preparedness measures. Also, a large-scale climate phenomenon such as ENSO may predict ecological processes better than weather-based models by integrating the effects of multiple weather variables that interact in complex ways^53^.

Our El Niño-based infectious disease risk assessment for coming months requires some caveats. The development of El Niño events cannot be predicted with complete certainty. However, we expect predictability to be higher for this near-term forecast (versus, for example, a 1-year-ahead forecast). Predictability even at longer lead-times has been favorable without a high false-alarm rate during 1857-2003^54^. Additionally, in September 2013 a model distinct from NOAA’s predicted a 75% chance that El Niño would develop in late 2014^55^.

A limitation to ENSO-based infectious disease predictions is the complex relationship between ENSO the and dynamics of certain diseases. The relationship has been strong and consistent for some diseases, such as RVF in East Africa^5^^,^ 7. However, nuanced analytical approaches applied to long epidemiological time series have identified inconsistent ENSO associations with incidence of cholera in Bangladesh^22^ and dengue in Thailand^17^; found no systematic relationship between ENSO and dengue incidence in Mexico, Puerto Rico, or Thailand^56^; and uncovered a previously undetected association with plague in the western United States^44^. Factors that could modify ENSO’s impacts include local or other large-scale climatological processes, as well as unrelated phenomena that influence disease dynamics, such as waning or persisting human immunity.

Our observations do not attempt to predict disease outbreaks or even elevated transmission risk, but instead highlight the occurrence of current and future ENSO conditions that might influence environmental conditions that influence the dynamics of certain diseases (Table 2, Figure 5). The linkages we describe between climate and disease transmission are based on many years of observations of teleconnections between specific persistent weather/climate anomalies and specific vector-borne and water-borne disease outbreaks in specific regions.

We encourage public health organizations to take steps to mitigate El Niño impacts, such as increased incidence of infectious disease, in areas likely to be affected. While some measures are specific to certain diseases, preparations should include risk communication to the public and relevant sectors; vector and public health surveillance to detect increased disease activity; and planning for surge capacity to respond to increased disease activity. In particular, we emphasize the need for public health monitoring as El Niño develops to improve understanding of regional health effects and facilitate development of early warning systems.

## Competing Interests

The authors have declared that no competing interests exist.
